# Ephemeroptera of Canada

**DOI:** 10.3897/zookeys.819.26411

**Published:** 2019-01-24

**Authors:** Luke M. Jacobus

**Affiliations:** 1 Division of Science, Indiana University Purdue University Columbus, 4601 Central Avenue, Columbus, Indiana 47203, USA Indiana University Purdue University Columbus Columbus United States of America

**Keywords:** aquatic insects, biodiversity assessment, Biota of Canada, Ephemeroptera, mayflies

## Abstract

Thus far, 335 currently valid species in 82 genera and 21 families of mayflies (Ephemeroptera) have been documented from Canada, remarkably representing a little more than half of the combined species richness of Canada, Mexico and the USA. The current known species richness for Canada represents an increase of 11.3% as compared to that reported in 1979. Species richness is greatest in the families Heptageniidae (83), Baetidae (76) and Ephemerellidae (45). A total of 328 DNA Barcode Index Numbers (BINs) are available for Canadian mayfly species. The greatest net gains anticipated for future species tallies are for Baetidae (25), Heptageniidae (10) and Leptophlebiidae (10). A total of 66 more species overall is anticipated for Canada, with greatest gains potentially coming from lentic habitats across Canada and from far eastern and far western areas in general. However, even metropolitan areas should not be overlooked for the potential of discovery.

“Great strides” have been made in our understanding of the Canadian mayfly fauna since Lehmkuhl (1979) used this phrase in his review of the status of this order as part of the larger work by Danks (1979). At the time, Lehmkuhl (1979) indicated that there was “no comprehensive treatment of the Canadian fauna.” That situation has since been remedied with a continually updated Canada species list that has been available online since 1995 (McCafferty 1996, McCafferty and Jacobus 2018). Ecological and biological information was summarized by Waltz and Burian (2008) and others in previous editions of that work. Peters (1988) provided a biogeographic discussion of the Canada fauna that remains largely applicable today. McCafferty et al. (1990) discussed the status and needs of mayfly systematics in North America north of Mexico, including Canada. A history of mayfly science in Canada was summarized by McCafferty and Randolph (1998) and further reviewed by McCafferty (2001).

Thus far, 335 currently valid species in 82 genera and 21 families have been documented from Canada, remarkably representing a little more than half the combined species richness of Canada, Mexico and the USA (McCafferty and Jacobus 2018). Two species described from Canada are considered nomen dubia, and thus, they are not included in species counts (McCafferty and Bae 1992, Jacobus and McCafferty 2007). At least 15 of the species documented from Canada have a Holarctic distribution (e.g., Kluge 1980, Novikova and Kluge 1987, Kluge and Novikova 2011, Bauernfeind and Soldán 2012, Kjærstad et al. 2012, Savolainen et al. 2014, Cordero et al. 2016). Eight of the Canadian species have geographic distributional ranges that extend south to Central America (McCafferty and Jacobus 2018). Ten of the Canadian species are not yet known outside Canada, but the number of truly endemic species probably is much lower, because most of these ten have been collected near the southern border and likely occur in the United States also (Jacobus 2018). Low endemism in Canada is not surprising, considering the recent glacial history of Canada and therefore the large degree to which the current fauna reflects dispersal and post-glaciation recolonization events (Peters 1988, McCafferty and Randolph 1998). No non-native species are known from Canada (Randolph et al. 2002).

The current known species richness for Canada represents an increase of 11.3% as compared to that reported in 1979. The families with the largest net increase in species numbers are Heptageniidae (10), Caenidae (6) and Baetidae (5). Ameletidae probably has nearly as great an increase as Heptageniidae (Zloty 1996, Zloty and Harper 1999), but current and 1979 species numbers could not be compared due to the way data were classified in the 1979 report (see footnote 7 in Table 1).

**Table 1.** Census of Ephemeroptera in Canada.

Taxon^1^	No. species reported in Lehmkuhl (1979)	No. species currently known from Canada^2^	No. BINs^3^ available for Canadian species	Est. no. undescribed or unrecorded species in Canada	General distribution by ecozone^4^	Information sources
**Suborder Pisciforma^5^**						
**Superfamily Baetoidea**						
Baetidae	71	76	87	25	most ecozones	McCafferty and Waltz 1990, Lugo-Ortiz and McCafferty 1998, Randolph et al. 2002, Jacobus and McCafferty 2005, McCafferty et al. 2005, 2010, Guenther and McCafferty 2008, McCafferty 2011a, Burian and Myers 2011, Jacobus and Wiersema 2014, Cruz et al. 2017, Webb et al. 2018
**Superfamily Siphlonuroidea**						
Acanthametropodidae **^6^**	1	1	0	1	Boreal Plains, Prairies	Lehmkuhl 1976, McCafferty 1991a
Ameletidae **^7^**		23	18	5	nearly all ecozones	Zloty and Harper 1999
Ametropodidae	2	2	1	0	central & western Boreal & Taiga ecozones	Jacobus 2013
Metretopodidae	3	3	3	1	most ecozones	Berner 1978, Engblom et al. 1993, Kluge 1996
Siphlonuridae **^7^**	35	20	12	1	nearly all ecozones	Traver 1935, Kondratieff and Voshell 1981, Provonsha and McCafferty 1982, McCafferty and Edmunds 1997, Newell and Anderson 2009, Burian 2017
**Suborder Setisura^5^**						
**Superfamily Oligoneurioidea^5^**						
Isonychiidae **^8^**	5	6	4	1	most ecozones south of taiga	Kondratieff and Voshell 1984
Oligoneuriidae	1	1	0	1	Prairies	Edmunds 1951
**Superfamily Heptagenioidea**						
Arthropleidae **^9^**	1	1	0	0	Taiga Shield, Boreal Shield, Hudson Plains, Atlantic Maritime, eastern Boreal Plains	Ide 1930, Wang and McCafferty 1995
Heptageniidae **^10^**	73	83	82	10	nearly all ecozones	Burian et al. 2008, Webb and McCafferty 2008, 2011
Pseudironidae **^11^**	1	1	0	0	Boreal Plains, Prairies, western Boreal Shield	Pescador 1985
**Suborder Carapacea**						
Baetiscidae	7	6	2	0	most ecozones south of Taiga Plains and Taiga Shield	Pescador and Berner 1981, Baumann and Kondratieff 2000, Webb and McCafferty 2003
**Suborder Furcatergalia**						
**Infraorder Lanceolata**						
**Superfamily Leptophlebioidea**						
Leptophlebiidae	24	27	34	8	nearly all ecozones	Traver 1935, Allen 1973, McCafferty 1992, Burian 2000, Tiunova and Kluge 2016, McCafferty et al. 2017
**Infraorder Scapphodonta**						
**Superfamily Potamanthoidea**						
Potamanthidae	3	4	0	0	Atlantic Maritime, Mixedwood Plains, extreme southern Boreal Shield	Bae and McCafferty 1991
**Superfamily Ephemeroidea**						
Ephemeridae **^12^**	11	9	8	2	most ecozones south of Arctic	McCafferty 1975, 1994
Palingeniidae **^13^**	1	1	0	0	southern Boreal Shield, eastern Prairies	McCafferty 2004
Polymitarcyidae	3	3	4	0	Prairies, Boreal Plains, Boreal Shield, Mixedwood Plains, Atlantic Maritime	McCafferty 1975, 1994, Molineri 2010
**Infraorder Pannota**						
**Superfamily Caenoidea**						
Caenidae	10	16	15	4	nearly all ecozones	Provonsha 1990, Sun and McCafferty 2008
Neoephemeridae	1	1	0	0	Mixedwood Plains	Schmude et al. 2012
**Superfamily Ephemerelloidea**						
Ephemerellidae	44	45	50	5	nearly all ecozones	Jacobus and McCafferty 2008, Jacobus 2010
Leptohyphidae **^14^**	4	6	8	2	most ecozones, except for Arctic, Taiga Cordillera, Taiga Plains, and Boreal Cordillera	Alba-Tercedor and Flannagan 1995, Wiersema and McCafferty 2000, 2005, Baumgardner et al. 2003, Baumgardner 2009
**Total**	**301**	**335**	**328**	**66**		

**^1^**Mayfly higher classification is far from resolved. This classification is a synthesis based on Sun et al. (2006), Ogden et al. (2008, 2009: Figs 1, 7), Prisecaru et al. (2014), Miller et al. (2018). **^2^**Based on Mayfly Central species list for North America (McCafferty 1996, McCafferty and Jacobus 2018). **^3^**Barcode Index Number, as defined in Ratnasingham and Hebert (2013). Only those BINs that include Canada specimens are included in counts. Counts accurate as of 30 April 2018. ^4^See figure 1 in Langor (2019) for a map of ecozones. **^5^**Taxon known to be paraphyletic, even when considering only North American fauna, north of Mexico. **^6^**Recorded in 1979 as Siphlonuridae, subfamily Acanthametropodinae. **^7^**Due to changes in family-level classification, the 1979 report of species in the subfamily Siphlonurinae now applies to species in the current families Ameletidae and Siphlonuridae. For simplicity, the pooled 1979 total of 35 species has been inserted for Siphlonuridae and nothing has been entered for Ameletidae. **^8^**Recorded in 1979 as Siphlonuridae, subfamily Isonychiinae. **^9^**Recorded in 1979 as Heptageniidae, subfamily Arthropleinae. **^10^**Today this family excludes taxa that were reported as Arthropleinae and Pseudironinae in 1979. Thus, the number of species reported herein for 1979 includes only those reported for Heptageniinae and Anepeorinae. **^11^**Recorded in 1979 as Heptageniidae, subfamily Pseudironinae. **^12^**Recorded in 1979 as Ephemeridae, subfamily Ephemerinae. **^13^**Recorded in 1979 as Ephemeridae, subfamily Pentageniinae. **^14^**Recorded in 1979 as Tricorythidae.

The current family and genus classifications used here for the Ephemeroptera of Canada are quite different from those that were hypothesized nearly 40 years ago, as reflected in the work of Lehmkuhl (1979). Current family-level classifications in North America, and Canada in particular, primarily reflect the work of McCafferty (1991b), with subsequent revisions or significant review by Landa and Soldán (1985), Peters and Peters (1993), Wang and McCafferty (1995), and McCafferty (2004). Higher classification remains tentative and contentious in many cases, as reviewed by Ogden et al. (2009), and the higher classification followed here borrows from various sources (see footnote 1 in Table 1) to best reflect the most recent phylogenetic hypotheses (Jacobus and McCafferty 2006, Ogden et al. 2009: fig. 7, Miller et al. 2018).

The classification of species into genera also has changed markedly in the last 40 years. The family Baetidae has seen the greatest number of changes, with multiple new genera described, several more recent generic revisions, and some species going back and forth between genera several times. McCafferty and Waltz (1990) provided a summary of these changes up to that point, along with some of their own, and that work marks the starting point for Baetidae references cited in Table 1; the reference list is long but not exhaustive, providing a strong starting place for understanding the current generic classification applied to the species found in Canada. Heptageniidae also has seen considerable changes in generic classification of species, and most of the current genus-level systematics for Canada are reflected in Webb and McCafferty (2008). Sun and McCafferty (2008) made significant changes to genera of the subfamily Brachycercinae in the family Caenidae, and Jacobus and McCafferty (2008) most recently revised the generic classification of Ephemerellidae. The classification of species into genera across the order remains inconsistent and somewhat contested globally (e.g., Bauernfeind and Soldán 2012, Jacobus 2015, 2016).

The number of new species described and named from Canada has been relatively few since 1979, but the most notable gain has been in the genus *Ameletus* Eaton (Ameletidae) (Zloty 1996, Zloty and Harper 1999). In contrast to new species descriptions, a remarkable number of species synonyms have been proposed for the Canadian fauna, and these are reflected in the complete species synonymies given by McCafferty and Jacobus (2018). However, it should be noted that new evidence, especially from DNA barcoding, challenges many of these concepts of highly variable species, and some of the current concepts of single species may be split into multiple species after more research is completed (Webb et al. 2012). Recently collected mayfly specimens from Canada have played an important role in the generation of regional DNA barcode libraries (Ball et al. 2005, Zhou et al. 2009, 2010, Webb et al. 2012) and discovering trans-Atlantic species distributions (e.g., Kjærstad et al. 2012, Savolainen et al. 2014, Cordero et al. 2016).

While a list of species for Canada that reflects current species concepts has been maintained online for over 20 years (see above), only coarse geographic distributions have been indicated there, with Canada divided into eastern, western, and far northern regions, essentially following those indicated by Edmunds et al. (1976: 50) and McCafferty and Waltz (1990: fig. 1). McCafferty and Randolph (1998), however, provided a comprehensive listing of species for each of the Canadian territories and provinces at that time. Subsequent notable contributions for each include the following for Alberta (Webb and McCafferty 2003, McCafferty et al. 2004, 2012, McCafferty 2009, Webb et al. 2012), British Columbia (Jacobus and McCafferty 2001, McCafferty et al. 2012), Manitoba (Flannagan et al. 2001, Jacobus and McCafferty 2001, McCafferty et al. 2004, McCafferty 2009, Zhou et al. 2009, 2010, Webb et al. 2012, Kjærstad et al. 2012), New Brunswick (Jacobus and McCafferty 2001, McCafferty 2009, Webb et al. 2012, Burian 2017), Newfoundland and Labrador (Lomond and Colbo 2000, McCafferty 2009, 2011b, Webb et al. 2012, Burian 2017), the Northwest Territories (Randolph and McCafferty 2001, Bowman et al. 2010, Gorski et al. 2015, Burian 2017, Giberson and Burian 2017), Nova Scotia (Jacobus and McCafferty 2001, McCafferty et al. 2004), Nunavut (Randolph and McCafferty 2001, Giberson et al. 2007, McCafferty 2011a, Burian 2017), Ontario (McCafferty et al. 2008, McCafferty 2009, Webb et al. 2012, Klubertanz 2016b), Prince Edward Island (Jacobus and McCafferty 2001), Quebec (Jacobus and McCafferty 2001, Randolph et al. 2002, Burian 2017), Saskatchewan (McCafferty et al. 2004, Webb et al. 2004, 2007, McCafferty 2009, Miyazaki and Lehmkuhl 2011, Webb et al. 2012), and Yukon Territory (McCafferty et al. 2004, Giberson and Burian 2017). Other scattered reports of new provincial and territorial records can be found in a variety of sources, including those listed in Table 1. The general distributions detailed in Table 1 are based on Canadian ecozones, as are the general distributions given for other taxa in this series of papers. Published records of species, as reviewed above, were used to determine the ecozones inhabited by each family.

Beyond those already reported in the scientific literature, an additional 66 species of mayflies are expected to be added to the list of Canadian species (Table 1). This tally includes named species known to occur in adjacent parts of the USA that will be found predictably in adjoining Canada (L Jacobus unpubl. data, SK Burian pers. comm., JM Webb pers. comm.), and it includes known, but unnamed, species likely new to science, especially from the families Ameletidae, Baetidae, Caenidae (Brachycercinae), Ephemerellidae and Heptageniidae (Jacobus et al. unpubl. data). For most of these unnamed species, more material is needed to allow a final decision and to facilitate descriptions; others simply are awaiting completion of formal scientific descriptions. A couple of southeastern United States species likely to occur in Canada have been diagnosed tentatively, however, and given informal designations by McCafferty et al. (2017).

Lehmkuhl (1979) indicated that very few descriptions of larvae were “adequate for taxonomic purposes. Therefore the state of knowledge…[was]…not tabulated” at that time. Major advances in our knowledge of the larval stages have occurred since then (McCafferty et al. 1990), and in fact the larva is now the most commonly and widely studied metamorphic stage of mayflies. Morphological identifications of larvae of most species from Canada can be made using two recent workshop manuals (Jacobus et al. 2014, Jacobus 2017), both available by request, and the works cited therein. Jacobus (2017) relies heavily on integrating two recently published identification keys (Klubertanz 2016a, McCafferty et al. 2017). Specimens collected from Manitoba must be identified with all these sources cautiously, given the mix of eastern and western species in the province. Although the knowledge of larvae is generally good, certain taxa still require better description, and a variety of cryptic species complexes require resolution (Webb et al. 2012, 2018). Morphological identifications of mayfly subimagos and female adults to the species level are usually very difficult to impossible at the present, and morphological identification of male adults is possible only via the use of a variety of published literature and unpublished research notes, some of which remains unavailable outside private libraries.

Looking to the future, additional work is needed on lentic habitats throughout Canada because unusual new taxa continue to be discovered from lakes throughout North America (Hill et al. 2010, McCafferty 2011a). Northern Canada in general requires much more work before its fauna will be documented adequately (McCafferty et al. 1990, Cordero et al. 2016, Giberson and Burian 2017), but even less remote areas continue to yield new records of species (e.g., Klubertanz 2016b). Provinces such as Newfoundland and British Columbia have less documented species richness than might be expected, based on richness of surrounding areas (McCafferty and Randolph 1998, Meyer and McCafferty 2007, McCafferty et al. 2012) and may provide opportunities for considerable discovery that will add to species numbers, including new country records for Canada. Although our knowledge of the larval stage of mayflies has improved drastically since Lehmkuhl’s (1979) assessment, association of larvae with male adults should remain a priority, even in geographic areas that are considered easily accessible and mostly well known. The Ottawa and Montreal regions, for example, contain type localities for species still unknown as larvae (e.g., McDunnough 1925).
